# The deuce-ace of Lassa Fever, Ebola virus disease and COVID-19 simultaneous infections and epidemics in West Africa: clinical and public health implications

**DOI:** 10.1186/s41182-021-00390-4

**Published:** 2021-12-30

**Authors:** Nnabueze Darlington Nnaji, Helen Onyeaka, Rine Christopher Reuben, Olivier Uwishema, Chinasa Valerie Olovo, Amarachukwu Anyogu

**Affiliations:** 1 Department of Microbiology, University of Nigeria, Nsukka, Nigeria; 2 School of Chemical Engineering, University of Birmingham, Edgbaston, Birmingham, B15 2TT UK; 3 German Centre for Integrative Biodiversity Research (iDiv), Halle-Jena-Leipzig, Leipzig, Germany; 4 Oli Health Magazine Organization, Research and Education, Kigali, Rwanda; 5 Clinton Global Initiative University, New York, USA; 6 Faculty of Medicine, Karadeniz Technical University, Trabzon, Turkey; 7 Department of Biochemistry and Molecular Biology, School of Medicine, Jiangsu University Zhenjiang, Zhenjiang, 212013 Jiangsu People’s Republic of China; 8 School of Biomedical Sciences, University of West London, London, W5 5RF UK

**Keywords:** COVID-19, Ebola virus disease, Lassa Fever, West Africa, Epidemics, Pandemic

## Abstract

Globally, the prevailing COVID-19 pandemic has caused unprecedented clinical and public health concerns with increasing morbidity and mortality. Unfortunately, the burden of COVID-19 in Africa has been further exacerbated by the simultaneous epidemics of Ebola virus disease (EVD) and Lassa Fever (LF) which has created a huge burden on African healthcare systems. As Africa struggles to contain the spread of the second (and third) waves of the COVID-19 pandemic, the number of reported cases of LF is also increasing, and recently, new outbreaks of EVD. Before the pandemic, many of Africa’s frail healthcare systems were already overburdened due to resource limitations in staffing and infrastructure, and also, multiple endemic tropical diseases. However, the shared epidemiological and pathophysiological features of COVID-19, EVD and LF as well their simultaneous occurrence in Africa may result in misdiagnosis at the onset of infection, an increased possibility of co-infection, and rapid and silent community spread of the virus(es). Other challenges include high population mobility across porous borders, risk of human-to-animal transmission and reverse zoonotic spread, and other public health concerns. This review highlights some major clinical and public health challenges toward responses to the COVID-19 pandemic amidst the deuce-ace of recurrent LF and EVD epidemics in Africa. Applying the One Health approach in infectious disease surveillance and preparedness is essential in mitigating emerging and re-emerging (co-)epidemics in Africa and beyond.

## Introduction

COVID-19, Lassa Fever (LF) and Ebola virus disease (EVD) are arguably the most prevalent infectious diseases in West Africa. The coronavirus SARS-CoV-2, which causes COVID-19, was first detected in December 2019 in Hubei Province, China [1, 2] and has since spread to all continents [3]. Egypt became the first African country to report a positive case of COVID-19 (14th February 2020), followed by Algeria on 25th February and then Nigeria on 27th February [4].

COVID-19 is spread from person to person via respiratory droplets and through direct contact with surfaces and other fomites [5]. Initial findings in China revealed a case fatality ratio (CFR) of 2.3% [2]. However, the World Health Organization (WHO) proposed a CFR of 4.91% in their 1st July 2020 report, based on 10,357,662 confirmed cases worldwide and 508,055 recorded fatalities [3]. Thus, the public health effects of COVID-19 are directly tied to the disease’s spread.

LF is an acute viral infection and a viral hemorrhagic fever (VHF) caused by the Lassa virus, a single-stranded RNA virus in the Arenaviridae family [6] of animal origin with symptoms comparable to malaria and COVID-19 in the early stages [7]. LF was initially identified in Nigeria in 1969, following the deaths of two missionary nurses in Lassa, Borno State. The disease is endemic in West African nations, including Sierra Leone, Liberia, Guinea, and Nigeria, where 300,000 to 400,000 cases are reported each year, with approximately 5000 deaths [8]. Mastomys rats shed the Lassa virus in their droppings and urine, and the virus is usually transmitted to humans through inhalation or ingestion. Direct contact with the droppings and urine of mastomys rats, from eating polluted food, touching soiled objects, or coming into contact with open incisions or sores, can result in infection [9]. The mortality rate for hospitalised patients varies between 5 and 10% [10]. Lassa Fever can be transmitted from person to person through bodily fluid contact, but not as quickly as EVD or coronavirus [7].

The Ebola virus disease (EVD) originally arose in two simultaneous outbreaks in 1976, the first in Nzara, South Sudan, and the other in Yambuku, Democratic Republic of the Congo (DRC). The latter occurred in a village near the Ebola River, which gave rise to the disease’s name [11]. The 2014–2016 EVD outbreak in West Africa was the largest since the virus was discovered in 1976. The disease began in Guinea and spread across borders to Sierra Leone and Liberia. EVD is a serious, frequently fatal illness in humans, despite its rarity. The virus is transmitted from wild animals to humans and spreads via human-to-human transmission. The typical fatality rate for EVD cases is roughly 50%. In previous epidemics, CFRs ranged from 25 to 90% [11].

The largest epidemic of EVD, and the first outside of Central Africa, occurred between 2013 and 2016. In the epidemic’s early stages, cases were primarily concentrated in three West African countries, Guinea, Liberia, and Sierra Leone. However, EVD soon spread to other countries and continents, including Mali, Nigeria, Senegal, Spain, Italy and the United States [12, 13]. Before 2013, isolated outbreaks of EVD had been reported, but these had been mainly confined to rural areas in Gabon, DRC, and Uganda [14]. The 2013 epidemic was the first time EVD spread to densely populated urban areas, providing conducive conditions for transmission. After a global, coordinated response, all affected countries were declared EVD free by 2016. By this time, 28,600 cases and 11,325 deaths had been reported. However, in 2021, outbreaks of EVD have been reported in three West African countries, the DRC, Guinea, and Cote d’Ivoire. It is significant to note that this is the first outbreak of EVD in Cote d’Ivoire in more than 25 years [15].

The emphasis on COVID-19 in Nigeria is a significant disadvantage in the fight against LF. While states have increased their awareness campaign to combat Lassa Fever, the issue is that the same individuals saddled with the responsibilities of fighting it are also dealing with the dreaded COVID-19 pandemic. The focus is now primarily on COVID-19, which is influencing how people react to Lassa Fever. In addition to the COVID-19 pandemic and its consequences, instances of Lassa Fever in the African population have been reported to decrease due to reluctance to contact clinics, which leads to unreported cases—all of which can contribute to a stealthy outbreak in West Africa [16]. When they finally visit the hospital, it is almost too late [17].

In the meantime, LF and EVD remain persistent in West Africa and COVID-19 cases and deaths are increasing across the continent [18]. The burden of a mixed epidemic could result to grave danger for a collapse of Africa’s convulsive healthcare system and to a large extend social life [19]. Strict lockdowns as a result of COVID-19 jeopardised EVD control efforts in DRC, resulting in ineffective EVD surveillance and contact tracing [20]. Furthermore, personnel responsible for managing EVD were redirected to containing the COVID-19 issue. This decrease in workforces, attention and measures has put the continuation of effective surveillance of EVD and LF in jeopardy. Africa has a narrow window of opportunity to avoid this catastrophic tragedy. Before the COVID-19 pandemic, many African countries were already struggling to achieve the criteria for quality healthcare systems in the WHO ‘Six pillars’ framework. These descriptors, including (a) leadership/governance, (b) healthcare financing, (c) health workforce, (d) medical products and technologies, (e) information and research, and (f) service delivery, were designed to evaluate the process and progress of strengthening healthcare systems and achieving sustainable development goals focused on good health and wellbeing [21]. However, African healthcare systems’ core challenges have been identified as inadequate human resources, insufficient healthcare systems, and poor leadership and management [7, 22].

Following the 2014 EVD outbreak, African governments provided governance by drafting an emergency preparedness and biosecurity strategy, as well as overseeing and coordinating disaster preparedness activities like was done by the Lagos State, government [23]. Constructing a comprehensive surveillance system, training critical personnel, and establishing a Biosafety Level 3 laboratory and biobank all helped to boost emergency response capabilities. Significant policy emphasis has been placed on the need to detect and contain emerging diseases that could lead to pandemics, as well as to raise and sustain investment in preparedness and health capacity [24]. The gradual expansion of molecular testing across the continent has been remarkable, with the emergence of trusted voices and leadership from national public health institutes such as the five regional Centers for Disease Control, the Nigeria Centre for Disease Control, and the apex Africa Centre for Disease Control adding technical rigour and improving response quality by guiding political leaders to make better public health decisions.

Even though global collaboration is improving, as can be seen in the activities towards containing the EVD epidemic, Africa has recognised the importance of robust health systems capable of responding to public health emergencies and providing high-quality health care to all citizens. Accordingly, African countries have formalised their commitment towards achieving Universal Health Care for all by 2030 [18]. However, more substantial governmental commitment to health is required for Africa to achieve this goal.

This review highlights efforts towards responding to the challenges of managing the deuce-ace of Lassa Fever and EVD outbreaks in the face of the COVID-19 pandemic in Africa. It also discusses potential solutions.

## The trio-epidemics

The occurrence of epidemics that risk public health is not strange in Africa. Apart from the current COVID-19 pandemic and its associated public health concerns, there has also been a simultaneous upsurge of the Lassa Fever epidemic and the resurgence of EVD within the African continent. Nigeria, the most populous African country, has cases of Lassa Fever reported in 32 of its 36 states, with an increasing number of cases occurring during the second wave of the COVID-19 pandemic [25]. Furthermore, recent reports show higher cases of Lassa Fever mortality during the second wave COVID-19 compared with the first wave of COVID-19 [25–27]. While COVID-19 surges through Africa, the WHO declared an EVD outbreak in DRC on 1st June 2020 [28]. This 11th EVD outbreak emerged from the Equateur Province, with a cluster of cases spreading from Mbandaka and then gradually to 11 of the 17 DRC Provinces. In less than 3 months, a total of 102 EVD cases were confirmed, and 44 deaths were recorded [29]. Since then, EVD spread northwards to Kivu Province, with confirmed cases reported on 7th February 2021 by the DRC Ministry of Health. Kivu province was the most severely affected province during the 10th EVD epidemic, which occurred between 2018 to May 2020, having the highest number of cases recorded throughout the history of EVD [30].

The COVID-19 pandemic, EVD and Lassa Fever co-epidemics have significant commonalities in their presentation and public health response. The concomitant occurrence of these epidemics may undoubtedly result in grave and prolonged clinical and public health implications across the African continent. Improved personal hygiene, isolation, and social distancing have proven to effectively prevent the community spread of COVID-19 and mitigate the total collapse of the weakened healthcare systems in Africa. However, widespread underdevelopment and poverty in Africa have left many densely populated, impoverished, and remote areas without access to essential sanitation services, including potable water supplies. Furthermore, apart from concerns about the impact of the co-circulation of the trio of COVID-19, EVD and Lassa Fever upon the African healthcare system, little or no work has been carried out on the possibility of widespread co-infections within the population. Although it has not been established whether such co-infections will lead to more significant clinical impact and severity, research must focus on the possibility of this emerging trend. However, previous reports suggest that co-infections with different pathogens may result in complex, severe and unpredictable pathophysiological consequences [31–33].

For example, in dengue fever endemic regions, COVID-19 and dengue fever co-infections have been widely reported [34–38]. Similar observations have been noted for dengue and flu [31, 32], and these have often been associated with greater severity. Unfortunately, early, prompt and proper laboratory diagnoses of infectious diseases are not routinely conducted in Africa and even when carried out, results are usually delayed. Therefore, clinicians mostly rely on the clinical manifestation of diseases to proceed with healthcare. However, COVID-19, EVD and Lassa Fever share similar clinical manifestations, especially at their early presentation [27]. While some clinical signs may point to COVID-19, EVD or Lassa Fever in case series, at the individual patient scale, the imperfect positive predictive value and clinical variability do not guarantee a diagnosis of certainty [33]. Therefore, on presentation of cases, patients must be explored for these three diseases simultaneously. In the context of co-epidemics constituting the co-circulation of three fatal viruses, it is crucial to enable a combined simultaneous diagnosis of COVID-19–EVD–Lassa Fever, especially for ambulatory patients.

In Africa, the number of COVID-19 deaths increased by more than 40% in the second week of July 2021 compared to the preceding week. New and more rapidly spreading variants, such as the highly transmissible Delta form, are occurring in an increasing number of African countries, with less than 2% of the continent fully vaccinated [39]. Cases increased at an accelerated rate in June 2021 as Africa began facing a fast-surging third wave of COVID-19. Africa registered an additional one million cases in June 2021, the shortest period it has ever taken to add another million cases. Daily cases peaked at 28 per one million people during the second wave, and the rate is currently about 33 per one million populations during the third wave [39].

Presently, the total active cases of Lassa Fever in Nigeria are 318 in 14 states, with 2298 suspected cases in 32 states. The number of reported cases of Lassa fever began increasing again as the country struggled to contain the spread of the second wave of the COVID-19 pandemic [25]. Since the COVID-19 second wave began in January, more people have died of Lassa Fever in Nigeria [26].

According to the Africa Center for Disease Control (ACDC), there has been no new cases or fatalities from EVD in Guinea since their latest brief of 8th June 2021. In total, 23 cases of EVD have been reported, with 12 fatalities (CFR: 52%) and ten recoveries. To date, all known contacts have been followed up. In Guinea, a total of 10,873 people have been immunised against the Ebola virus. Nevertheless, member States bordering Guinea are on high alert. Since the emergence of EVD in Guinea, Sierra Leone has reported 57 alerts [40].

## Current challenges facing responses to Lassa Fever and Ebola virus disease in Africa during the COVID-19 pandemic

### The overburdened healthcare system

The COVID-19 pandemic began immediately after the peak of Lassa fever in Nigeria, resulting in a COVID-19 and Lassa Fever crisis [41] that needed to be tackled by the already limited number of medical personnel in the country. Nigeria has 74,543 registered physicians, but only 40,000 are estimated to be practising in a country which has a population of 201 million. The physician to patient density is 4 doctors for every 10,000 patients and 16.1 midwives and nurses for every 10,000 patients. This is less than the WHO recommendations of 1 doctor for every 600 patients and the critical threshold of 23 doctors, midwives, nurses per 10,000 patients [42]. There was an increase in the number of confirmed Lassa Fever cases during epidemiological week 9 (early March) of 2020 when the first laboratory-confirmed case of COVID-19 was identified in Nigeria. The early emergence of Lassa Fever in 2020 was prompted by the increased number of cases from the 2019 Lassa Fever epidemic [27]. In 2020, there was a 46.3% increase in the 2019 number of confirmed cases. In 2019, the number of confirmed cases were 796, and by 2020 the number of confirmed cases were 1165 [43].

As expected, the COVID-19 pandemic has a variety of devastating public health and socioeconomic effects over the world, culminating in a reduction in epidemiological control of various infectious illnesses, including zoonotic diseases [44–46]. In the fight against Lassa Fever, the focus on COVID-19 represents a significant disadvantage. While African nations have ramped up their public awareness campaigns to combat Lassa fever, the problem is that the same people saddled with the responsibility of fighting Lassa fever are simultaneously coping with the dreaded pandemic. Much attention is now on managing and combating COVID-19 [17], with LF control mostly ignored [41]. Cases of LF have been believed to be decreasing due to reluctance to contact clinics, which leads to unreported cases, all of which contribute to a quiet outbreak [16]. This situation poses a significant health risk, as the number of cases and deaths attributed to LF continues to rise each year, and healthcare personnel are particularly at risk [43, 47].

Before the 2021 COVID-19 outbreak, Aborode et al. [48] predicted an increase in Ebola incidences in Africa as a result of COVID-19, as several Ebola community engagement activities were halted. Strict lockdowns jeopardised Ebola control efforts, resulting in ineffective Ebola monitoring and contact tracing [20, 49]. The national Ebola response leadership was made responsible for managing the DRC’s COVID-19 response; as a result, multiple Ebola response staffs were moved to containing the COVID-19 outbreak. This decrease in measures, attention, and workforces put the continuation of effective EVD surveillance in jeopardy [50].

### Africa’s fragile health systems and medical professional shortages

Like every other continent, Africa has been affected by the ongoing pandemic, and the continent’s pre-existing healthcare infrastructure is a significant disadvantage [41]. Healthcare systems in Africa may be overwhelmed as a result of the broad spread of COVID-19. There are few critical care beds, fewer clinicians with the training to manage critical patients with complex respiratory needs, limited medical and personal protective equipment [51]. In addition, there are no ventilators in the public health system [52].

Compared to European and East Asian countries, skilled healthcare workers are in poor supply, falling 60% below the UN’s minimum threshold [41]. In Nigeria, internal and international migration of medical practitioners has posed a significant challenge to public health systems; it exacerbates already weakened healthcare systems, widening the global health inequalities gap [53]. Nigeria loses tens of millions of dollars per year by training medical doctors who then leave the country [54]. Medical doctors leave some African countries, especially Nigeria for high-income countries because of poor pay, irregular salary payments, poor working conditions (extra hours due to insufficient staff, lack of diagnostic facilities), and a lack of social amenities, which make it challenging to ensure a good standard of living [55]. These factors contribute to Africa having the lowest density ratios of medical professionals to the population in the world [56].

Medical professional shortages contribute to unequal access to health care within and between countries and inferior population health outcomes [57]. This brain drain has proved a significant disadvantage in tackling the COVID-19, LF, and EVD. Sub-Saharan Africa has only 1 to 5% of ICU beds per capita [41]. Over $160 billion has been made available for the COVID-19 response in East, Central, West, and Southern Africa to date, with 80% coming from public and philanthropic sources and 20% coming from private sources [41]. In addition, access to treatment, late presentation, and a high prevalence of untreated non-communicable diseases are the most important variables determining the increased mortality risk of these diseases [58].

### Misdiagnosis as a result of shared epidemiological and pathophysiological features

One of the biggest obstacles in the fight against LF is the similarity of its symptoms which include fever, headache, muscle aches, fatigue, sore throat, nausea, diarrhoea, vomiting [59–61] with those of many other common diseases including EVD and even COVID-19 [62]. This makes misdiagnosis possible with severe consequences and an increased risk of co-infection at the onset of both illnesses. Inadequate diagnosis of COVID-19 and other viral diseases due to overlapping symptoms is a problem because early detection is critical for effective treatment. In addition, many people are still opposed to vaccines to combat the virus and its spread [62].

### African economy fragility and widespread poverty

According to the World Bank, the International Poverty Line refers to those who earn less than 1.25 US dollars per day and consequently live on the very edge of existence. The United Nations Development Program (UNDP) metrics in its Human Development Index (HDI) to evaluate poverty in Africa and other countries indicate that Africa is severely poor. African countries are consistently ranked bottom in the United Nations’ yearly report on human development [63].

A look at the economic challenge of the COVID-19 pandemic has shown that Africa is highly vulnerable to the global economic upheaval induced by COVID-19. For example, in Nigeria, this is due to a sharp drop in oil prices. Oil accounts for more than 80% of exports, one-third of bank credit, and half of the government revenue [64]. Takyi and Bentuum-Enin [65] also found that the stock market performance in some African countries, including Mauritius, Ghana, Cote d’Ivoire and Nigeria were impacted negatively. The World Bank estimates that 40% of Nigerians (83 million) are poor, with another 25% (53 million) classified as vulnerable. This vulnerable population may slip into poverty due to the COVID-19 pandemic [66]. The magnitude of the health impact of COVID-19 is determined by the duration and domestic spread of the outbreak, whereas oil prices determine the economic impact.

In contrast to many rich countries of the West, most Africans rely on day-to-day labour for food. Many people are involved in small scale farming and informal trading to make ends meet as there is a high rate of formal unemployment, and so, with the implementation of lockdowns, thousands lost their jobs resulting in an increasing population with no reliable income source. Many people were caught between a rock and a hard place of staying home and facing hunger or going out and risking infection. The lockdown came with closures of food produce markets and other informal economic sectors such as the street vendors, causing a reduction in per capita income, a decline in value of supply chains, increase in debts to contain the pandemic, closure of schools, aviation and shipping industries and pressure on fiscal policies ultimately resulting in economic recessions.

In addition, poverty, to a significant measure, fuels cross-border migration, as individuals travel every day in search of work or food. West Africa has a high rate of people mobility over very permeable boundaries and has been noted to be seven times that of the rest of the globe [67]. The response and control of these infectious, viral diseases are hampered by difficulties caused by population mobility. Tracing cross-border contacts is also challenging. Although populations freely transcend porous borders, epidemic responders do not [67].

### African wildlife and human interactions

Infectious illness outbreaks are linked to the disruption of the human–animal–environment interaction [68]. More than 75% of newly emerging infectious illnesses are zoonotic, transmitted from animal hosts to humans [69]. Many zoonotic illnesses abound in Africa, from endemic zoonosis like brucellosis and leptospirosis to neglected zoonoses like rabies and onchocerciasis to emergent zoonoses like anthrax, yellow fever, Ebola, and Lassa Fever [68]. Increasing urbanisation and deforestation are all important anthropogenic drivers of developing zoonoses. Rapid migration from rural to urban regions frequently results in high-density slums with poor housing, limited access to clean water, and inadequate sanitation. In addition, the overburdened infrastructure of regional transit centres for air, land, and sea transport, such as Lagos (Nigeria), Freetown (Sierra Leone), and Dar es Salaam (Tanzania), offers ideal conditions for the spread of Ebola virus disease [70].

Disease transmission is aided by deforestation, which causes wildlife such as rodents and local fruit bats to migrate elsewhere in search of food, bringing various dangerous infections such as the Lassa virus, malaria parasites, and the Lyme disease bacterium [71]. Animal husbandry has also developed to suit Africa’s growing demand for animal protein. The exploitation of wildlife and the growth of wet markets to feed the bushmeat and bird consumption culture have increased interaction with livestock, allowing animal illnesses to spread to humans [68]. Bush burning for subsistence farming has also driven animal populations from their habitats in several West African and East African countries, resulting in seasonal surges in the frequency of zoonotic illnesses [72].

## Preventive and control measures

### The One Health approach in mitigating current epidemics

The increase in the incidence of zoonotic infections such as EVD and LF in Africa, severe acute respiratory syndrome (SARS) and middle east respiratory syndrome (MERS) in Asia, and COVID-19 globally. The current coronavirus pandemic has severe consequences for health, the economy, and society [73]. The loss of natural ecosystems brings humans in closer contact with animals that may transmit viruses that have never been seen before. As a result, the only way to effectively avoid localised epidemics and global pandemics are to consider human, animal, and environmental health as an entity, as the One Health concept provides [74].

One Health is a collaborative problem-solving strategy that aims to secure the wellbeing of humans, animals, and the environment on a local, national, and global scale. The One Health concept builds on existing capabilities while also bringing together disciplines and sectors to provide additional health advantages. Increased cross-sectoral coordination can aid in promoting science-based decision-making, the reduction of unnecessary duplication among the sectors responsible for human, animal, and environmental health, and the more effective handling of external variables impacting disease burdens [75].

The application of the One Health approach against LF will aid in gaining better insights into the zoonotic risk of this disease to humans as the ecology of LF will be better understood. Furthermore, identification of the different Lassa fever virus LASV strains and lineages harboured by already recognised rodent species is fundamental to the ecological understanding of LF [76]. As a matter of fact, with the aid of molecular analysis, a broad regional picture of which LASV lineages are distributed across key rodent populations within West Africa have been identified [77]. This has helped in identifying rodent-borne areas and potential targets for a future outbreak of Lassa fever.

**Fig. 1:**
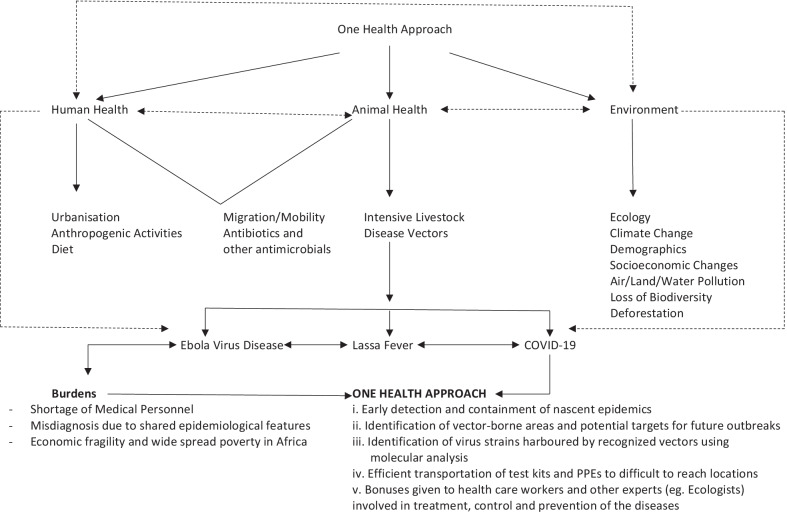
A summary of One Health implementation especially in the potential burden of three observed diseases

For effective control of EVD in Africa, adequate knowledge of the wildlife ecology about disease transmission is crucial, just like in LF. Many of these infectious diseases that have caused epidemics and pandemics such as influenza, MERS, LF, EVD, and COVID-19 have animal origins and are thus zoonotic diseases. The increased association of humans and animals enhances the possibility of the transmission of these diseases. Therefore, an all-inclusive data collation and analysis could be employed for the effective control of these diseases. This will aid in setting up an efficient intervention strategy that encompasses the forest and wildlife therein and other animals and humans that come in contact with them. Thus, with the One Health approach (Fig. 1), the overwhelming morbidity and mortality connected to zoonotic diseases could be prevented, and in time, human lives would be saved, and treatment costs would be significantly reduced [78].

### Genomic sequencing, surveillance and monitoring

A well-coordinated public health system for detecting and controlling emerging illnesses necessitates an effective collaboration between health care providers, academic health centres, and the traditional public health system. Physicians must send samples to competent laboratories to confirm the diagnosis and report the results to the public health system for effective infectious disease surveillance [79].

African countries have focused on rigorous surveillance and case-finding, using the Integrated Disease Surveillance and Response framework (IDSR) [80]. The IDSR system enables case-based and syndromic surveillance of 40 diseases, serving as a starting point for recognising, describing, and responding to COVID-19 community transmission.

The WHO is collaborating with governments in Africa to increase pathogen surveillance by genome sequencing in order to effectively detect and respond to variants of COVID-19. Africa CDC and WHO developed a COVID-19 sequencing laboratory network in Africa in 2020, this laboratory has produced more than 43,000 sequencing data to date [81]. Since the onset of the COVID-19 pandemic, African countries have also initiated efforts to integrate regular surveillance and genetic sequencing into national responses. South Africa, for example, discovered the highly transmissible Beta variant in December 2020, allowing the government to revise public health policies [82]. Beyond COVID-19, genome sequencing has the potential to improve public health and revolutionise responses to other important health problems [81].

### Preparedness for the next epidemic

It should be noted that some lessons learnt from the EVD epidemic have guided the actions taken since the coronavirus pandemic was first reported. For example, during the 2014 EVD epidemic, in Nigeria, the Emergency Operations Centre (EOC) was used as an Incident Management System (IMS) to coordinate the response and consolidate decision-making. The EOC is widely recognised for its assistance in the early containment of the EVD outbreak in Nigeria [83]. Many of the lessons learned from keeping the country free of Ebola [18].

Evidence suggests that pandemics have become more likely over the last century due to increased global travel and integration, urbanisation, changes in land use, and higher exploitation of the natural environment [24]. These tendencies are likely to persist and intensify. As a result, significant policy attention has been placed on the need to detect and contain nascent epidemics that could lead to pandemics and increase and sustain investment in preparedness and health capacity [24].

The gradual expansion of molecular testing across the continent has been remarkable. Countries (such as South Africa and Nigeria) are leveraging and integrating molecular laboratory diagnostic capabilities for specific disease programmes such as drug-resistant tuberculosis, Lassa Fever, and HIV to scale-up COVID-19 testing. While access to diagnostic reagents remains a critical challenge, some countries, such as Ghana and Rwanda, are pioneering pooled testing of COVID-19 samples [84, 85].

Across Africa, public health and social measures were also undertaken. For example, borders were closed, self-isolation for exposed people was implemented, and quarantine centres were formed. In addition, states and localities were placed on “lockdown”. As these public health initiatives reduce the rate of transmission, the healthcare system is projected to see fewer cases of critically ill individuals [86]. In addition, private hospitals are accredited by government authorities to fill gaps in the public health care system’s service provision by leading treatment facilities dedicated to caring for the most critically ill patients [58].

In response to the lockdowns and the difficulty of reacting to the pandemic, health and other non-health industries are piloting creative uses of technology and new methods of working. Drones, for example, are being used to transport test kits and samples to difficult-to-reach areas, reducing sample transport time from many hours to minutes; there is a surge in locally manufactured face masks; an explosion in locally produced soap and hand sanitisers; and training, meetings, and workshops have moved online. In addition, many governments have recognised the need to increase hazard compensation and provide insurance for front-line infection-control personnel with African corporations working together to provide cash and in-kind to support country initiatives, such as the $70 million donated by a Nigerian coalition [87].

Despite the obstacles to accessing testing in many countries, the open, regular, and honest transmission of testing findings within most African countries has kept the globe updated on disease progress. Following the West African Ebola outbreak, the emergence of trusted voices and leadership by national public health institutes such as the Nigeria Centre for Disease Control, the five regional Centers for Disease Control, and the apex Africa Centre for Disease Control has added technical rigour and improved response quality by guiding political leaders to make better decisions on public health [88, 89]. In addition, celebrities and social media influencers have joined public health specialists in encouraging individuals to exercise social distance [58].

Despite budget constraints, several African countries’ responses to COVID-19 have been fast, progressive, and adaptable [90]. These offer hope for Africa’s preparedness for the next epidemic.

## Conclusion

The deuce-ace of LF, EVD and COVID-19 simultaneous infections has hugely impacted African countries. Most West African countries have put in place mechanisms to combat these disease outbreaks. These efforts, however, are not without challenges. Africa must continue to step up efforts and solve issues to appropriately respond to the uncertainties that these infections present. This necessitates a unique approach to ensure a proactive response. In the region, good surveillance and monitoring and the One Health approach to controlling existing outbreaks should be prioritised. As we have seen, human, animal and environmental health are all intertwined, and the degradation of ecological systems has raised the overall risk of zoonotic disease outbreaks necessitating the immediate integration of the One Health Approach. The severe human, social, and economic consequences of COVID-19, LF and EVD should compel the International and West African community to work in synergy in these sectors to avert outbreaks, minimise the impacts of these diseases and be prepared for the possible emergence of new diseases. To address the problems of combating these viruses, it is very important to adopt interdisciplinary approaches such as education, vaccination and prevention, campaigns, improving hygiene and social distancing behaviours, and enhancing testing and management protocols. On a general note, given the enormous challenges confronting West Africa's healthcare systems, so many major reforms will be needed to guarantee a strong and resilient healthcare system in West Africa in the future. They include shifting the focus of health services from treating to preventive healthcare and keeping the African population healthy; giving local communities more power over medical resources; improving access to medical care through mobile technologies; and tightening restrictions on medications and medical gadgets, as well as enhancing their distribution; minimising reliance on international aid organisations in order to encourage the development of more reliable local supply; and expanding universal health insurance coverage to the impoverished West Africans.

## Data Availability

Not applicable.

## Abbreviations

COVID-19: Coronavirus disease 2019
WHO: World Health Organization
UN: United Nations
EOC: Emergency Operations Centre
EVD: Ebola virus disease
IMS: Incident Management System
UNDP: United Nations Development Program
HDI: Human Development Index A
LF: Lassa Fever
ACDC: Africa Center for Disease Control
SARS: Severe acute respiratory syndrome
MERS: Middle east respiratory syndrome
IDSR: Integrated Disease Surveillance and Response framework
VHF: Viral hemorrhagic fever
